# A versatile 4D capacitive imaging array: a touchless skin and an obstacle-avoidance sensor for robotic applications

**DOI:** 10.1038/s41598-020-68432-1

**Published:** 2020-07-13

**Authors:** Gege Ma, Manuchehr Soleimani

**Affiliations:** Engineering Tomography Laboratory (ETL), Department of Electronic and Electrical Engineering, University of Bath, Claverton Down, BA2 7AY UK

**Keywords:** Engineering, Health care

## Abstract

There are growing interests in the use of robots in collaborative environments with humans or other intelligent machines. Sensing the environment for which the robot is operating can be done in many ways, generally guided by skin-like sensors. Some of the skins are inspired by natural sensing in humans or other species. As humans, we use many of our senses, such as version, hearing, smell, and touch, to move around by avoiding colliding with other humans or objects. Different from humans, many other mammals also use whiskers as an additional sensor to help navigate around. In this paper, we demonstrate a touchless capacitive imaging-based sensor in the situation where the obstacles are in close vicinity to the robot. The proposed imaging system can sense the changes in areas near to the skin-like sensors by measuring the capacitances between the array of electrodes. A 4D sensing approach has been developed with the spatiotemporal Total Variation algorithm. The 4D operational mode gives sensors the time awareness that allows for dynamical responses and hence the better control of the robots. Several experiments are conducted to show the skin-like behaviour of this sensor by simulating various scenarios. The sensor shows the excellent ability to detect an object in its vicinity, where the depth is close to half of the planar sensor array size.

## Introduction

In recent years, the role of tactile sensing in robotics has received extensive increasing attention^1^. Tactile sensing is expected to widen the perceptual ability of robotics and to enhance their cognitive behaviour. There are growing bodies of work focusing on touch sensing techniques inspired by human-skin^2–6^. A vital function of the robotics skin is to guide the machines by sensing their environment and avoid unnecessary collisions. In the area of automation, a new direction has been raised, which requires the common work of humans and robots in the same area. Under such circumstances, providing a safe environment for the cooperation between human and machine is of great importance, e.g., ISO/TS15066 standard specifies the environment safety requirements for such cooperation. Achieving the sensing functionality of robots can be done via vision systems using a camera or other avoidance sensors such as ultrasound. Though the gold standard is still based on the vision system, it is critical to have the additional sensors for robots to detect an approaching human or an unwanted object. The reason for that is the vision system may suffer from the cases of occlusions, thus resulting in the reduce cooperation between robots, or even between robots and humans. Therefore, extensive researches are conducted in the developments and applications of various types of robotic sensing skin.

One important aspect of such a sensing skin is its ability to analyse the continuous data over time instead of obtaining and combining the static snapshot data. Taking the fabric-based piezoresistive skin as an example, this dynamical imaging method was shown to be more efficient in providing a more accurate and natural sense of contact and pressure^5^. However, the sensors made by piezoresistive fabric in^5^ needs direct contact to sense the touch pressure. A contactless skin, which enables to sense the spatial change within a short distance region, may offer a better option for collision avoidance in conjunction with the control set up of robotics. A whisker-like sensor was proposed in^7^ and was developed for 2D capacitive measurement in^8^, which is inspired by the fact that many animals use whiskers to sense their proximity to the other objects in the near environment. The authors in^9^ also demonstrate the principle of how an electric fish navigates their way by sensing the electrical fields. Therefore, detecting the dynamic electromagnetic field changes can be an efficient and accurate way to sense the environment change. In^10^, the authors review the role of robotic social interaction through skins, showing that dynamical sensing is key to provide natural interaction in social interaction. Thus, a 4D electrical capacitive tomography (ECT) is introduced in this study by numerous experiments to show its performance as a contactless and dynamical skin-like sensor.

ECT is an imaging modality which can visualize the permittivity distribution around a region through the capacitance measurements from a set of electrodes. A 3D planar array ECT system used in this work was developed in^11^ and further optimized in^12^. With the temporal image reconstruction method that directly interprets the correlations between images in successive data frames^13^, a 4D ECT system was extended^14^. The 4D tomographic approach based on the temporal reconstructive algorithm, is used to improve the imaging performance by taking multiple ‘frames’ of measurements for the same area and tracking changes. This method is well suited for our proposed applications and shows the advantages over traditional 3D imaging.

In this paper, a 4D touchless skin is shown by adaptation of a versatile sensing mechanism using the ECT sensing principle. The computational modelling of the electrical field and an advanced temporal total variation algorithm are briefly described in the method part, which followed by several examinations of this touchless skin-like sensor showing various of functionalities. Through the real-time data collection and time corrected analysis, we can use this contactless skin to achieve 4D imaging tasks.

## Measurement principle

**Figure 1:**
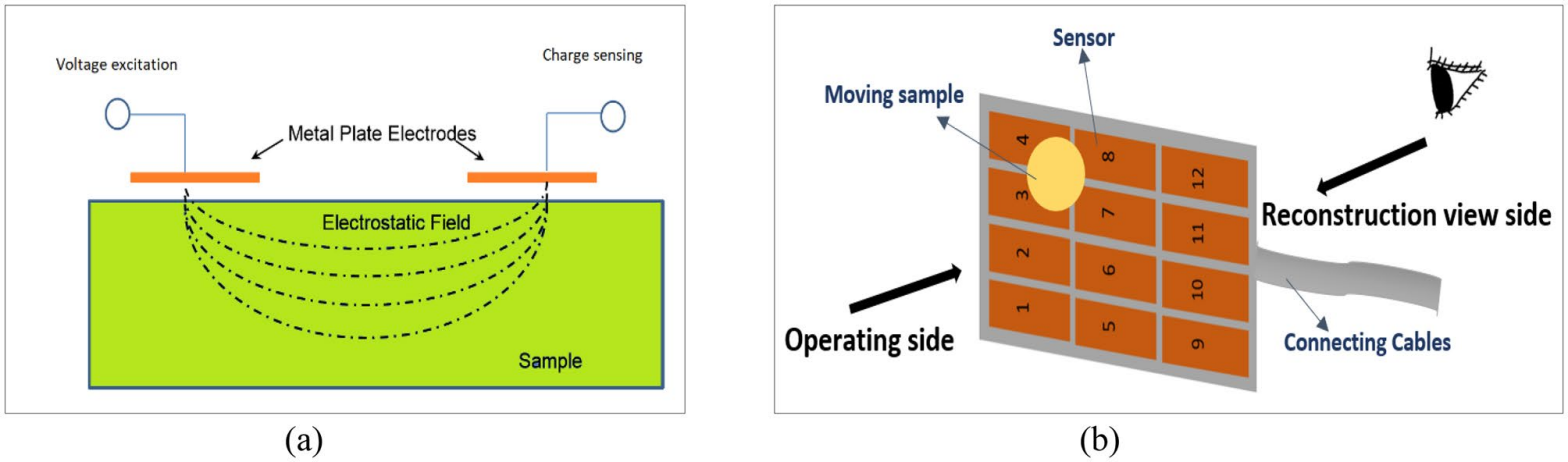
(**a**) The principle of the planar array ECT system. (**b**) The 12-electrode planar ECT sensor array.

Electrical capacitance tomography has been widely used in industry application working as a low-cost, real-time, non-destructive, and contactless imaging technique. The aim of ECT is to reconstruct the distribution of the dielectric materials with the permittivity contrast in the sensing range through the capacitance measurements of electrode pair^15^. A typical ECT system comprises an array of sensors, the data acquisition system which is used for collecting data from the sensors, and a host PC for data analysis and image reconstruction. The sensors can be built up in the circular array or planar array. In this work, a planar sensor array consisting of 12 same size conducting plates is used. Figure 1a shows the principle of the planar array ECT system and Fig. 1b is the diagram of the 12-electrode planar ECT sensor array used in this paper. After injecting a low-frequency AC voltage to a pair of electrodes, an electrostatic field is formed in the near spatial around the electrodes, with which the ECT sensors can detect the permittivity changes.

During each measuring process, the excitation voltage is applied to one of the electrodes, which is called the transmitting electrode, while all the other electrodes are grounded. The total charge on the receiving electrode is measured so that a capacitance value between the transmitter and the receiver can be calculated. This process will be repeated until all electrodes have acted as the transmitting electrode and as the receiving electrode in turn. For this reason, the number of independent measurements and the number of electrodes have the relationship given by $M=N\times (N-1)/2$, where *M* is the number of independent measurements and *N* is the number of electrodes.

## Method

In ECT, one needs to address the forward problem and the inverse problem to generate images. The forward problem is the process of evaluating the capacitances when the electric field is applied across the area of two electrodes. The inverse problem is the process of reconstructing the permittivity distribution through the capacitance measurements. The inverse problem can be solved after building the forward problem model.

A. Forward problem

The ECT system used here works in frequency of 1.25 MHz, so the dynamic electromagnetic field can be regarded as a quasi-static electromagnetic field. Using a low-frequency approximation to Maxwell’s equation, the electrical potential can be described as

1 $$\nabla \cdot \left(\varepsilon \nabla \phi \right)=0$$

where *ɛ* is permittivity and *ϕ* is electric potential. As the electric charge is the surface integral of electric flux through an area, the capacitance can be defined as

2 $$C= \frac{1}{V}{\oint }_{\Omega }\varepsilon \nabla \phi d\omega $$

where *V* is the excitation voltage, *Ω* is the surface area made up of elements *ω*. It reflects that the capacitance is a function of permittivity. During the forward modelling, a necessary process is to discretize the whole region of interest (ROI) and the electrodes by using the finite element method (FEM). When a small perturbation happens in permittivity $\Delta \varepsilon$, a change in capacitance measurements, $\Delta C$, can be obtained. The relationship between them can be then established by linearizing Eq. (2) through Taylor expansion:

3 $$\Delta C= J\Delta \varepsilon $$

where *J* is the Jacobian, which is essentially a ‘sensitivity distribution’ for each electrode pair showing how each capacitance measurement is affected by each voxel of the permittivity distribution.

B. Inverse problem

The inverse problem attempts to produce a permittivity distribution from the capacitance measurements with the Jacobian matrix. As it’s an ill-posed problem, the regularisation algorithm is generally added to tackle the problem. A simple method that can do this is Linear Back Projection (LBP), and it can be improved by adding regularisation terms as what Tikhonov Regularisation does^16^. A more advanced method for planar array ECT sensors is based on the total variation (TV) regularisation which allows a better depth detection^12^. In the case of the dynamical sensitive skin-like sensor application, we develop a 4D imaging algorithm which combines the temporal and spatial together, offering a great opportunity for the natural recovery of the test sample as it moves. Additionally, it has been shown that this algorithm can enhance the signal-to-noise ratio (SNR) performance of the ECT sensor^14^.

For the 4D ECT imaging sense, the dielectric permittivity can be described as $\varepsilon (x,y,z,t)$, where *x, y, z* are the spatial parameters and *t* is the temporal parameter. In the actual ECT experiments, we are dealing with a discrete number of spatial and time steps, which can be described by spatial and time resolutions. In dynamic ECT, with moving inclusions, the assumptions of the forward modelling Eq. (1) are still valid. Still, the expected permittivity difference would be a 4D object, which contains both spatial and temporal components. The spatiotemporal Total Variation Algorithm (STTV) to process the reconstruction work can be written as^17^:

4 $$arg\underset{{\Delta \varepsilon }}{{\min}}{\Vert {\nabla }_{x,y,z}\Delta \varepsilon \Vert }_{1}+{\Vert {\nabla }_{t}\Delta \varepsilon \Vert }_{1}, s.t. {\Vert \stackrel{\sim }{J}\Delta \varepsilon -\Delta C\Vert }_{2}^{2}\le \delta $$

where the first and the second term correspond to the isotropic spatial TV and temporal TV functionals, respectively. $\Delta \varepsilon$ represents a 4D dielectric distribution and $\stackrel{\sim }{J}$ is an augmented Jacobian operating on a frame-by-frame basis. The SSTV is solved based on the Split Bergman method and has been used to reconstruct dynamical images. The details of STTV are presented in^16^. The ECT image reconstruction in experimental data presented in the following section is carried out on a sensor array, that is rigid but, in principle, the sensor can be designed to be wearable and flexible. The work here is focusing on imaging obstacles, but the proposed sensors can be extended to identify objects in grips where the sensors can work better than the single plane. For applications of robotic grips, view^18^.

## Applications and results

In this part, experimental results are shown for various touchless skin scenarios with the 12-electrode planar ECT system. The evaluation of the proposed method and the performances of the system are demonstrated in three parts: (A) pressure mapping, (B) the functional performance for in-air handwriting, and (C) the obstacle detection, scanning and trajectory recording. The capacitance measurement data was collected by using the PTL 300E, an ECT device from the process tomography limited, PTL^15^. With the device, twelve channel capacitance measurements can be collected by using a pulsed excitation system, which allows capacitance values down to 0.0003pF to be resolved. This ECT System is capable to collect data in 100 frames/s rate, making it suitable as a rapid imaging device. The frame rate and the resolution of the capacitance measurements are critical not only for real-time imaging but also for capturing capacitances with significant variations in the planar setting. For all tests with 4D ECT, the multi-frame data were collected. And the reconstructed images shown in this work are the extractions from 4D videos which are the natural perception of a moving/dynamical object.

A. Pressure sensing

To evaluate the ability of the proposed method in the force and pressure mapping imaging, a number of experiments were accomplished with our ECT system. A deformable foam is used to create a simple pressure sensor together with the planar array capacitive plates.

**Figure 2:**
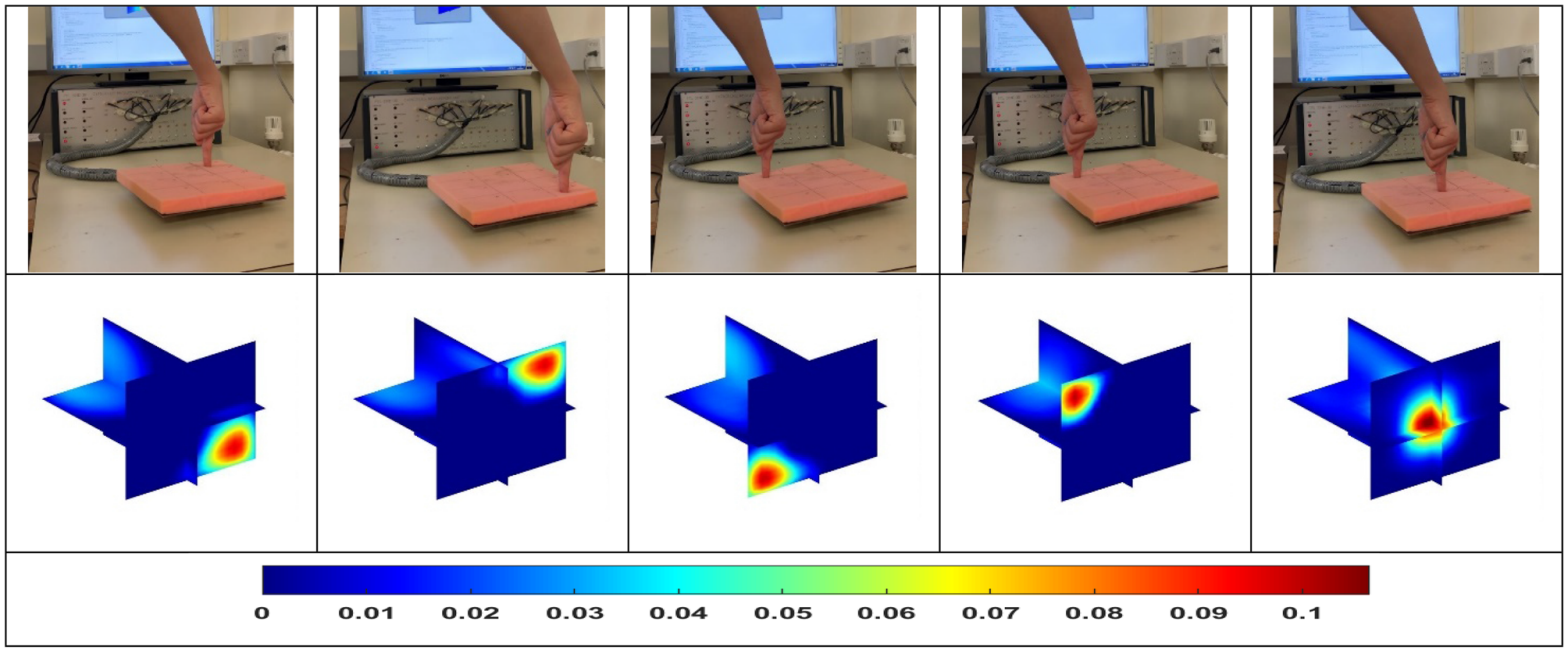
Single pressure point test.

Shown in Fig. 2, the single finger pressure test was carried out firstly with five different locations. The background data was obtained with no force applied as the reference, then the force was applied to the four corner points and the centre point step by step. During the test, the force applied to the foam at each location maintained as same as possible. From the reconstructed 3D images, it is clear to see the location of the pressure point. In addition, the scale of the colorbar represents the pressure force in a quantitative way. Needs to point out that all images are normalized into the same colorbar.

**Figure 3:**
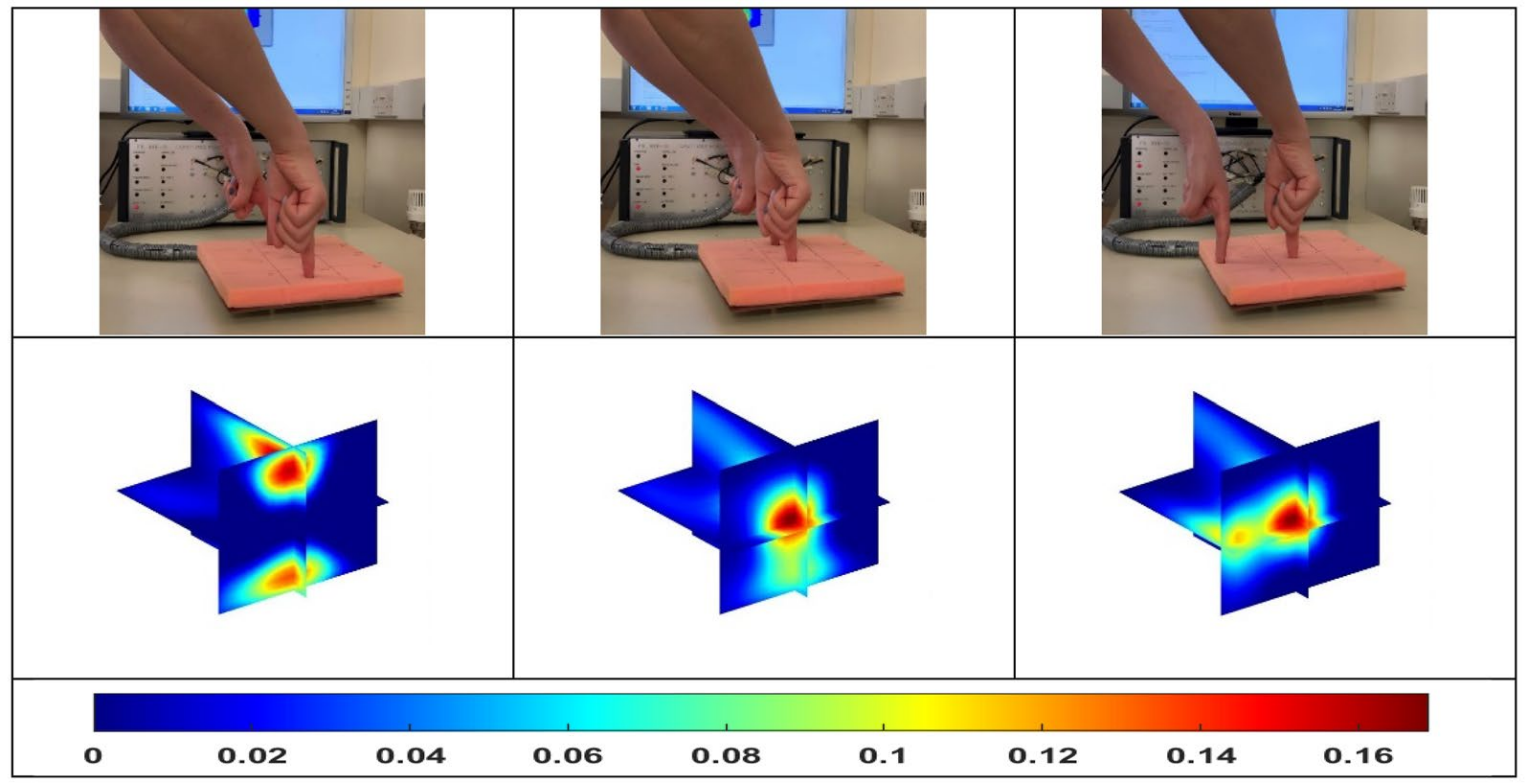
Double pressure points with uniform and non-uniform overall sensitivity.

**Figure 4:**
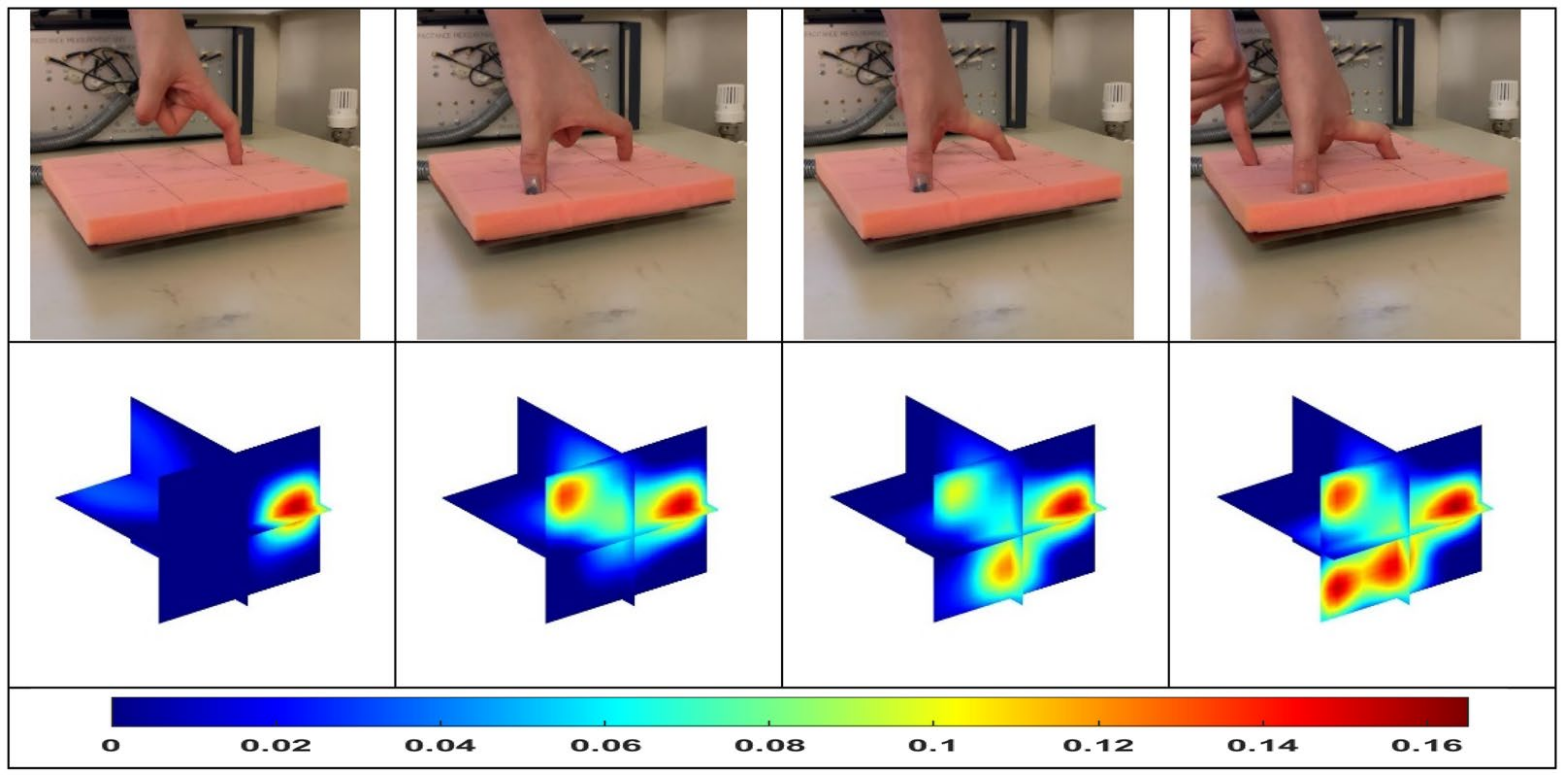
Multiple points of pressure: one, two, three and four points of sensing.

Following the single pressure point, the tests of double and even multiple pressure points were conducted with a similar approach. Figure 3 shows the experiment setup and the reconstructed 3D images for double pressure points. When the force was applied to both ends of the same horizontal line (the first column in Fig. 3), the reconstructed image shows a close value to the two pressure points. While when one pressure point is in the central area and the other one is in the edge area (the second and third column in Fig. 3), the results show a higher value for the point in the centre. This is true as the planar ECT has a higher sensitivity in the central area than that of the border area^12^. And the promising results illustrated in Fig. 4 shows that it is possible of this sensor, but not limited, to distinguish four points of pressure and contacts in an image.

**Figure 5:**
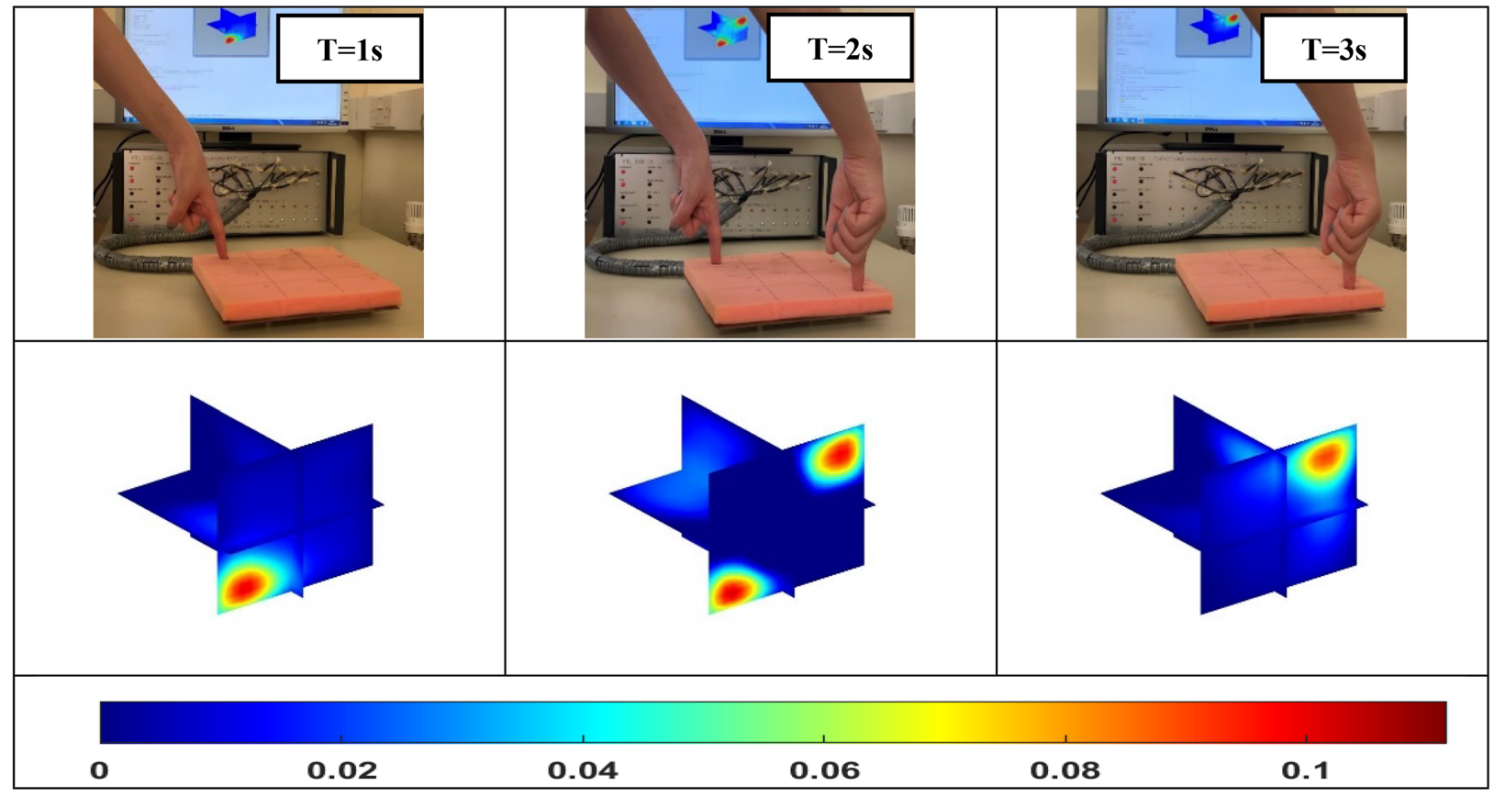
The dynamic change of pressure applied from one point to two points, and back to one point.

To explore the ability of 4D ECT to track the changes in pressure points in real-time, we conducted the experiments demonstrated in Fig. 5. The force was applied to the corner of the sensor array by a single finger firstly, then another pressure point was added along the diagonal line, and lastly, the first pressure point was removed. With the change of time, this 4D imaging system successfully tracks the change of pressure points.

**Figure 6:**
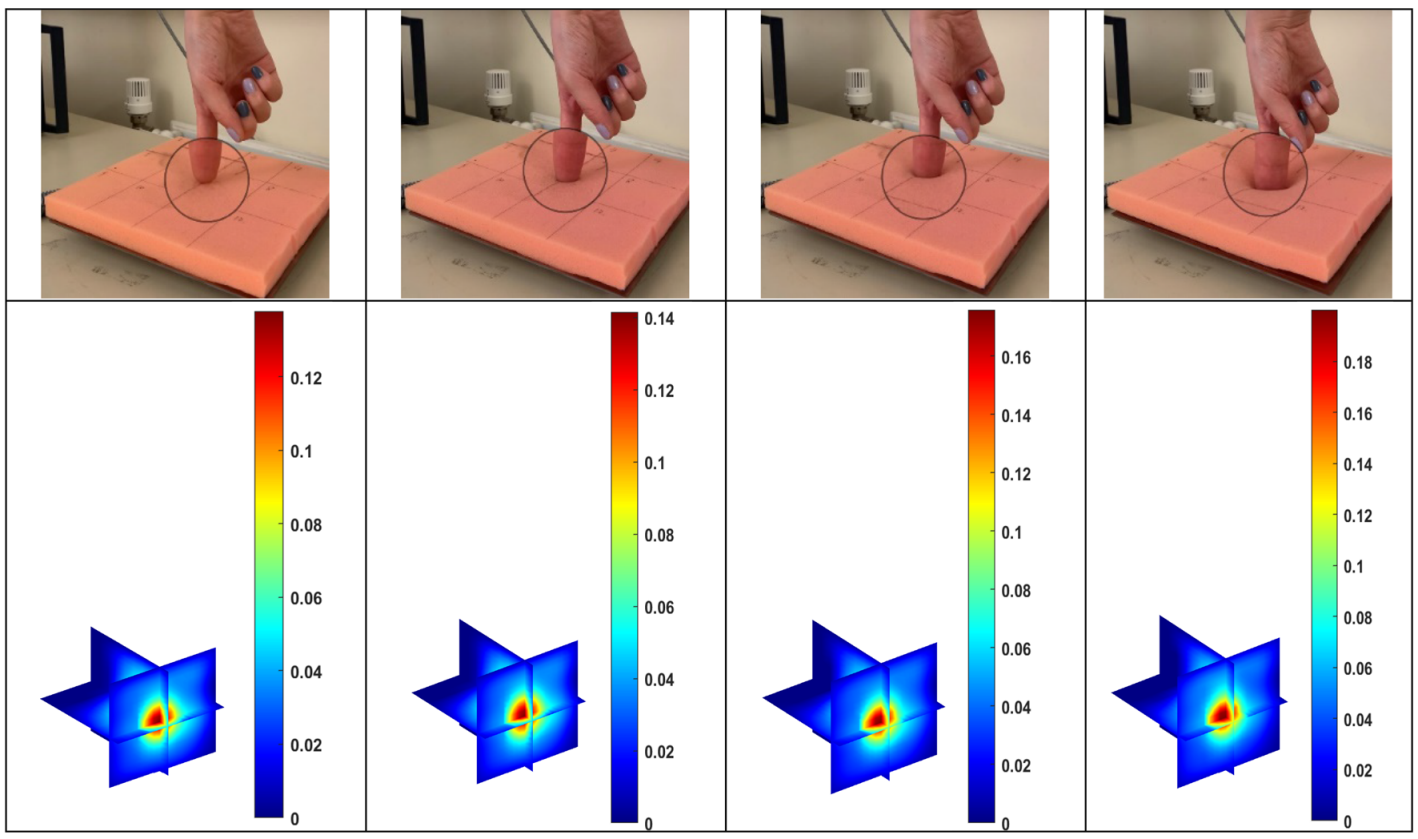
The Tests of pressure strength sensing**.**

And lastly, the experiments about the different pressure strength were tested, in order to investigate how the image scale shows a proportionality with respect to the applied pressure. From the left column to the right one in Fig. 6, there is a gradual increase in force. And this change can be clearly reflected through the corresponding colorbar scale, where the maximum image scale is changing from 0.12 to 0.14 to 0.16 to 0.18 from the left to the right.

B. Air hand drawing

**Figure 7:**
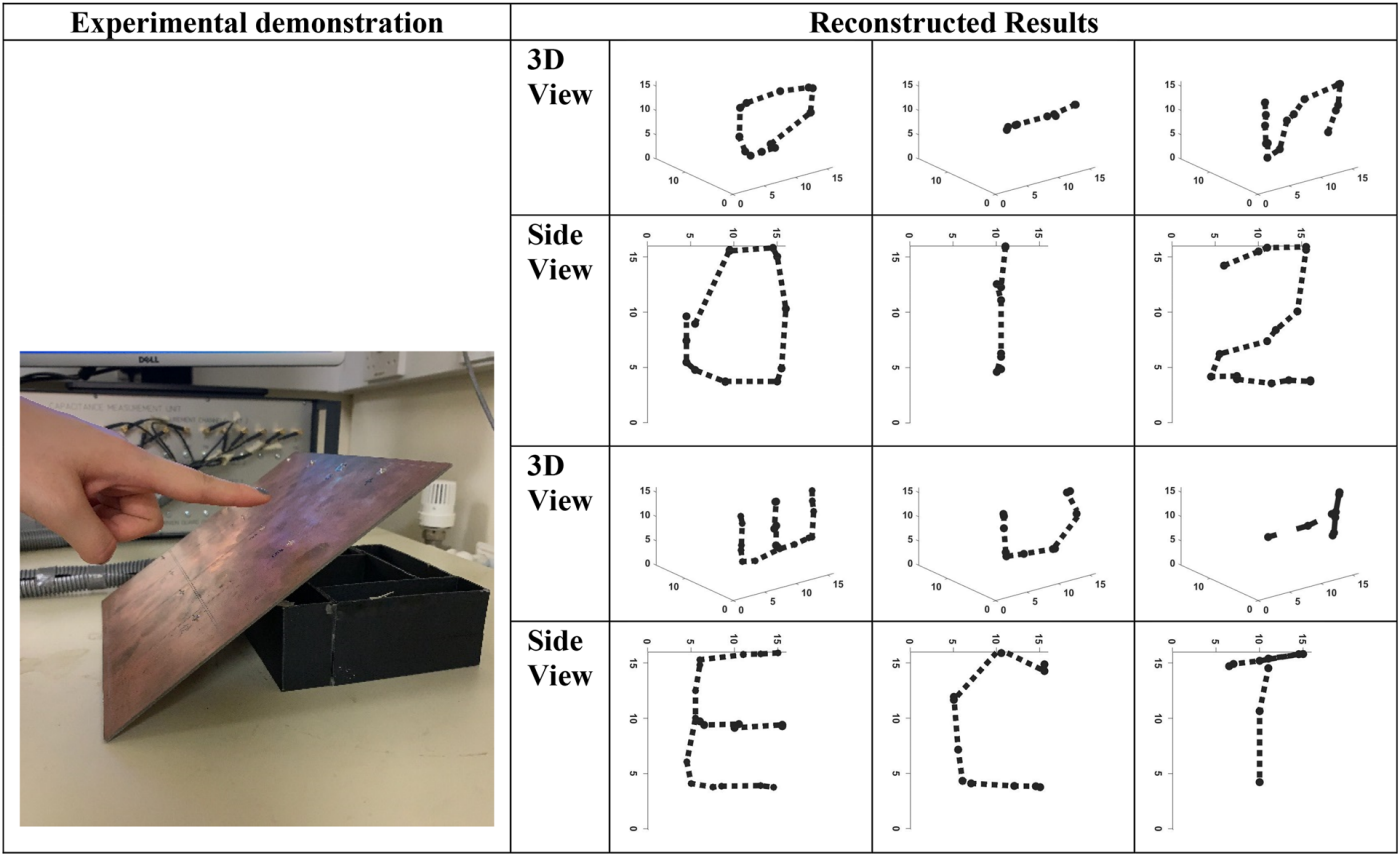
In Air handwriting (**a**) demo picture (**b**) reconstruction examples for 0, 1, 2, E, C, and T.

Although the skin is not necessarily a surface to write, such a capability shows the natural intractability of the proposed capacitive imaging. Figure 7 shows the in-air handwriting where many frames of capacitive sensor imaging combined in 4D fashion to deliver an output, which is the shape of letters or numbers. With the imaging device capable of 100 frames per second, the proposed sensor can provide an alternative medium for smooth communication through handwriting and hand drawing. The letter or number can be produced at a different distance away from the surface of the planar array sensor allowing for the real-time 4D handwriting.

C. Obstacle detection and trajectory mapping

**Figure 8:**
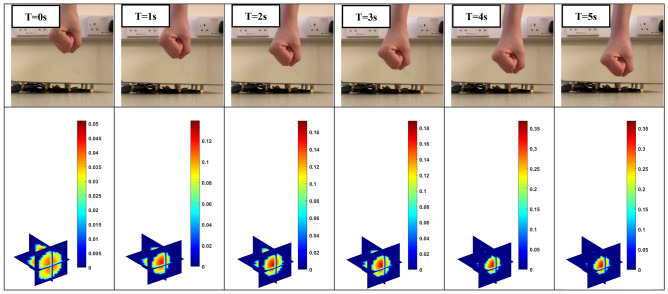
Position of obstacle as it approaches the sensor array (row 1); the reconstructed 3D images (row 2).

**Figure 9:**
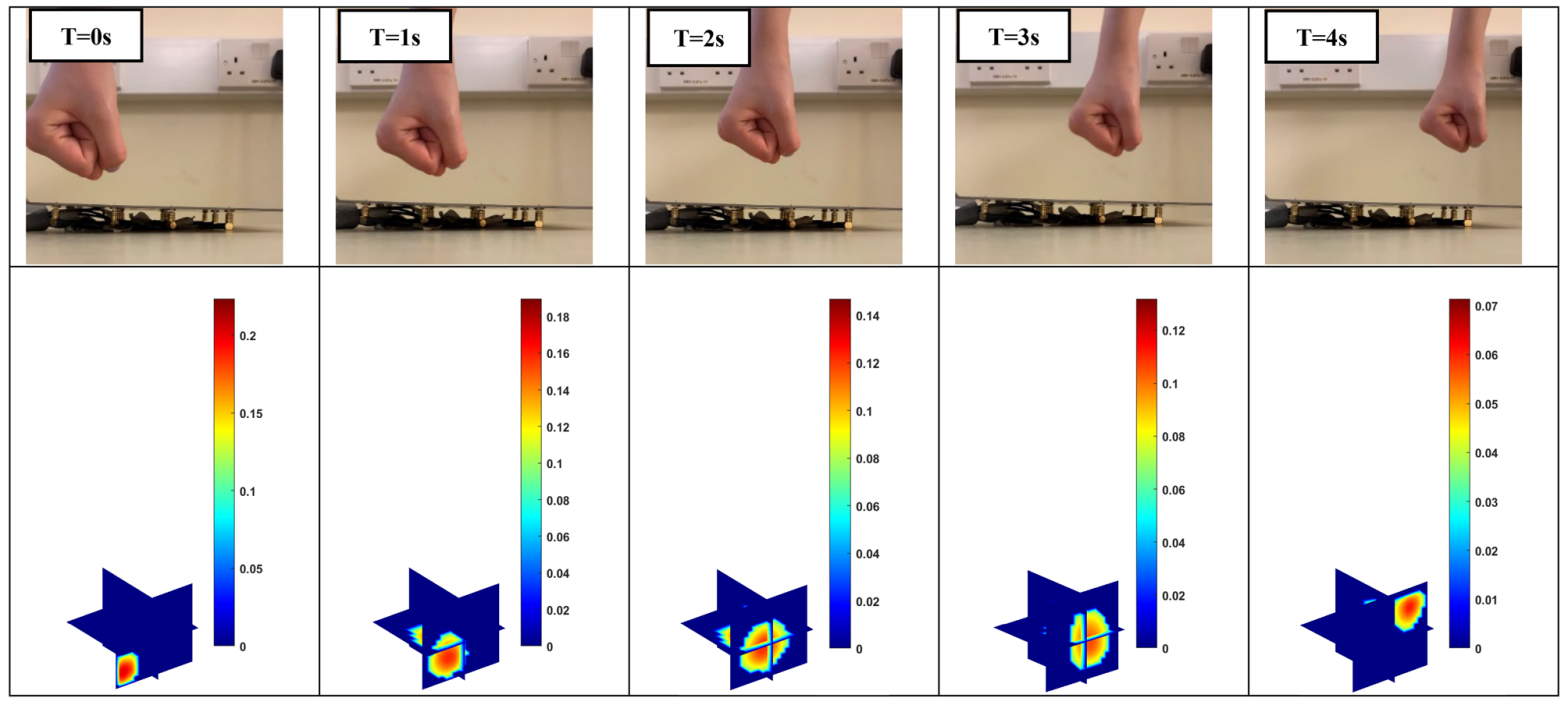
An obstacle moves along the diagonal direction of space (row 1); the reconstructed 3D images (row 2).

An ultimate use of artificial skin for robotic operation is the identification of the obstacles, which enables the robots to operate in an unexpected environment. The 4D ECT-based sensor here offers a great opportunity to develop such skin that can give a very high accuracy touchless sensing in short distances. Figure 8 shows the situation where the fist moves towards to the sensor array over time. This movement is again well reflected through the imaging color scale where the maximum values are changing from 0.06 to 0.37. Figure 9 shows the movement of the fist along the diagonal direction of space that is above the sensor array. As the obstacle moves away, the maximum scale of the imaging colour drops from 0.2 to 0.07. The reconstructed results confirm the ability of the 4D ECT-based sensor to detect and track object in real-time. One of the advantages of 4D mapping proposed here is that a predictive in-time model could be developed to control the movement of the robots based on the obstacle trajectory with time. For example, in Fig. 8, if the collision of the robot surface (skin) and the moving hand needs to be avoided, a predictive model could thoroughly analyse the situation that the object is approaching the sensor, and then further evaluate its moving speed based on the previous time step. Such additional and specific analysis are beyond scope of this paper.

**Figure 10:**
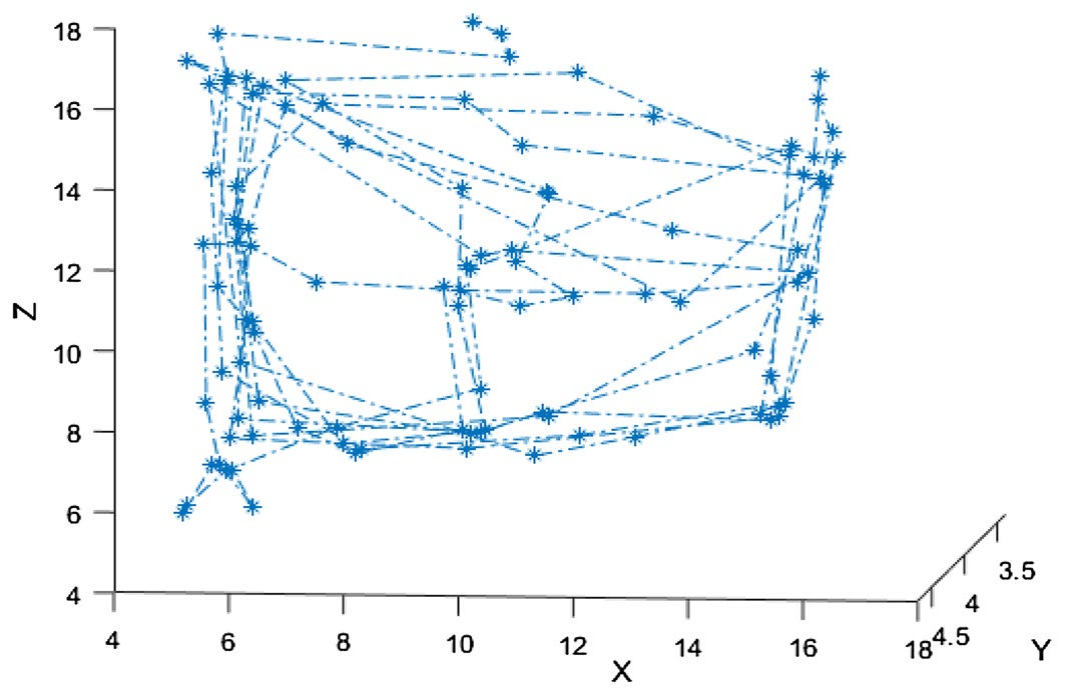
The path identification of an object moving from 100 location points.

The results shown in Fig. 10 is the position of the centre of the mass of the obstacle making a full movement path 100 data frames were collected when a hand was moving around the sensor in different direction and distances. The movements include moving along the diagonal direction, backward and forward. And the trajectory of the object movement was reconstructed using the 4D algorithm. Since the data from the sensor array is recorded in time steps, a full 3D velocity profile can be created giving rise to a full dynamical trajectory analysis.

## Discussion

This paper introduces and evaluates a new 4D contactless skin-like sensor. The performance of the proposed skin was analysed with the dynamical type data from a planar array ECT sensor. Besides, a method of 4D imaging algorithm was developed to account for the time-dependent behaviour of objects moving continuously in front of the sensors. An advanced ECT device was used in this study to show the imaging and the obstacle mapping principle. But a dedicated measurement system and the simplified low-cost hardware could be designed specifically if only the certain type of tasks is needed. The sensor enables to work as a pressure or force sensor if it is linked with a mechanically deformable medium. Mechanical performance of such a medium is not part of this study but can be optimised in future studies. In addition, the sensor works well for identifying the single and multiple objects by applying the slightest level of pressure or even no touch to our simple mechanical interface. There is a good quantitative correlation between the distance from the sensor and the imaging scale, the strength of pressure applied and the imaging scale, respectively. Calibration studies are needed for each specific case to make exact quantitative comparisons. We also demonstrated that this sensing technique can be used as a medium for more challenging communication tasks such as writing letters and words in the air. And more importantly, the obstacle detection and obstacle trajectory maps were also shown, which can be useful for working in an unpredictable environment and avoiding the collision. The sensor array used in this study are rigid, but the capacitive electrodes can be designed as a flexible sensor if needed. The system and method developed here can be versatile, covering many areas of robotics skin and obstacle tracking.
